# Laminopathies: what can humans learn from fruit flies

**DOI:** 10.1186/s11658-018-0093-1

**Published:** 2018-07-06

**Authors:** Marta Pałka, Aleksandra Tomczak, Katarzyna Grabowska, Magdalena Machowska, Katarzyna Piekarowicz, Dorota Rzepecka, Ryszard Rzepecki

**Affiliations:** Laboratory of Nuclear Proteins, Faculty of Biotechnology, University of Wroclaw, Fryderyka Joliot-Curie 14a, 50-383 Wroclaw, Poland

**Keywords:** Lamins, Lamin Dm, Lamin C, Nuclear lamina, Nuclear envelope, Laminopathy, *LMNA*, Fruit fly

## Abstract

Lamin proteins are type V intermediate filament proteins (IFs) located inside the cell nucleus. They are evolutionarily conserved and have similar domain organization and properties to cytoplasmic IFs. Lamins provide a skeletal network for chromatin, the nuclear envelope, nuclear pore complexes and the entire nucleus. They are also responsible for proper connections between the karyoskeleton and structural elements in the cytoplasm: actin and the microtubule and cytoplasmic IF networks. Lamins affect transcription and splicing either directly or indirectly. Translocation of active genes into the close proximity of nuclear lamina is thought to result in their transcriptional silencing. Mutations in genes coding for lamins and interacting proteins in humans result in various genetic disorders, called laminopathies. Human genes coding for A-type lamin (*LMNA*) are the most frequently mutated. The resulting phenotypes include muscle, cardiac, neuronal, lipodystrophic and metabolic pathologies, early aging phenotypes, and combined complex phenotypes. The *Drosophila melanogaster* genome codes for lamin B-type (lamin Dm), lamin A-type (lamin C), and for LEM-domain proteins, BAF, LINC-complex proteins and all typical nuclear proteins. The fruit fly system is simpler than the vertebrate one since in flies there is only single lamin B-type and single lamin A-type protein, as opposed to the complex system of B- and A-type lamins in *Danio*, *Xenopus* and *Mus musculus*. This offers a unique opportunity to study laminopathies. Applying genetic tools based on Gal4 and in vitro nuclear assembly system to the fruit fly model may successfully advance knowledge of laminopathies. Here, we review studies of the laminopathies in the fly model system.

**Electronic supplementary material:**

The online version of this article (10.1186/s11658-018-0093-1) contains supplementary material, which is available to authorized users.

**This article was specially invited by the editors and represents work by leading researchers.**

## Lamins

Lamins are evolutionarily conserved proteins, defined as class V intermediate filament (IF) proteins [1]. Typically, lamins are of the B- or A-type. All lamins share the same structure, independent of organism of origin: a central, α-helical rod domain flanked by an N-terminal head domain and a C-terminal tail domain [2–4]. The rod domain consists of four coiled-coil domains separated by flexible linkers [5]. The head domain is of variable length and contains several conserved motifs including a Cdk1 (cyclin-dependent kinase 1) site. The tail domain also contains several conservative motifs, including a PKC (protein kinase C) site, NLS (nuclear localization signal), a Cdk1 site and farnesylation motif (CaaX) site on the C-terminus. In lamin A, the C-terminal farnesylation motif with the farnesyl moiety is cleaved off when they reach the nucleus.

Lamins are nuclear proteins thought to be responsible for the structural organization of the nuclear envelope, nuclear lamina and chromatin in the metazoan nucleus [6]. They are also implied to play a direct or indirect role in chromatin organization [7], regulation of replication and transcription [8, 9], splicing [10], proper spacing of nuclear pore complexes, signaling, the connection between the nuclear skeleton and cytoplasmic skeletal structures [11], nuclear positioning [12–14], mechanosensing, and mechanotransduction [15–17].

**Fig. 1:**
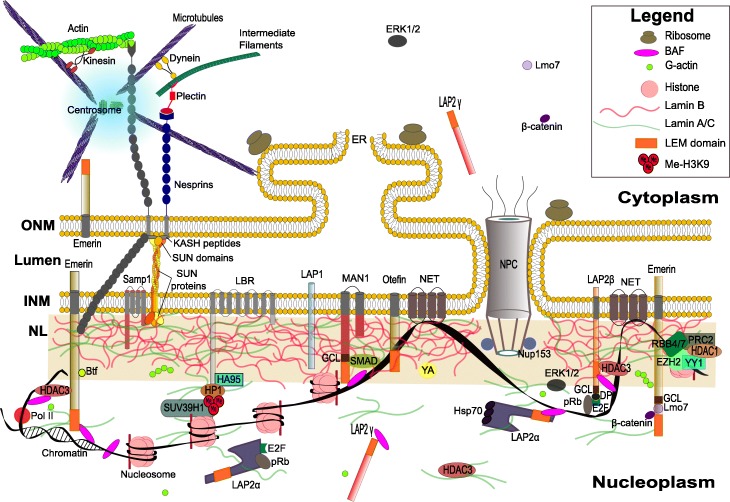
A simplified diagram of the interaction between the protein components of the nuclear lamina and the nuclear envelope with chromatin and the cytoskeleton. Only well documented proteins with high importance for biological functions are demonstrated. Please note that we included two fly-specific proteins (YA and otefin) along with the typical vertebrate proteins. ONM – outer nuclear membrane; INM – inner nuclear membrane; NL – nuclear lamina; NET – nuclear envelope transmembrane proteins; LBR – lamin B receptor; NPC – nuclear pore complex

Figure 1 demonstrates a simplified view of the interactions and relationships between lamins, LINC (linker of nucleoskeleton and cytoskeleton) complex proteins, the cytoskeleton and the major proteins of the nuclear envelope, nuclear lamina and chromatin. Based on the current state of knowledge on lamins, we may assume that a major role of lamins in vivo may be their function as a skeletal platform or hub, integrating many different signaling networks and signals. This includes mechanical signals and trafficking between the cytoplasm and nucleus. Simultaneously, they are responsible for mechanical support and protection for chromatin and the entire cell nucleus. Lamins participate in cellular mechanosensing and mechanotransduction through their direct link to the ECM (extracellular matrix) via the LINC complex, which directly interacts with cytoskeletal networks (F-actin, the microtubules/centrosome, and the cytoplasmic IF-filament proteins) connected to the ECM. Lamins regulate the organization of chromatin and modulate gene expression by providing a skeletal network for specific chromatin-binding proteins (BAF, HP1 and HDAC1–3) that interact with LEM-domain proteins, LBR, Samp1 and NETs. This integrates them into an interconnecting system at the nuclear lamina. Similarly, lamins regulate proper NPC distribution. On the outside face of nuclear envelope, lamins maintain a proper connection with cytoplasmic networks by positioning LINC complexes, which are fixed by interactions with lamins and other nuclear lamina proteins.

Note that LINC complexes are not the only connection between the karyoskeleton and cytoplasmic skeletal network. The nucleus is directly linked to centrosomes (microtubule-organizing centers, MTOCs) through direct interaction with proteins associated with the cytoplasmic face of NE (e.g., emerin) or indirectly through microtubule-binding proteins associated with NE.

So far, lamins were thought to be unique to metazoans, although there are several reports suggesting that lamin-like proteins may be present in plants and fungi. In most invertebrates, there is only a single lamin gene encoding B-type lamin, e.g., *C. elegans* has a single Ce-lamin. Note that Ce-lamin does not have the Cdk1 sites flanking the rod domain that are typical for the other analyzed lamins. Instead, PKC sites are used for depolymerization of Ce-lamin filaments mediated by phosphorylation. Some invertebrates may have more than a single lamin gene [18–21].

In the fruit fly, there are two lamin genes: one coding for lamin Dm, which is of the B-type, and one for lamin C, which is of A-type. In vertebrates, there are more lamin genes. In mammals, there are two B-types: *LMNB1* for lamin B1 and *LMNB2* for lamin B2 and B3. The latter arises by alternative splicing of the *LMNB2* gene, mostly in reproductive tissues. *LMNA* codes for two major splicing variant proteins: lamin A and lamin C. Minor products are lamin A (Δ10) and lamin C2, which is expressed in reproductive cells.

A much more complex system of genes for lamins exists in lower vertebrates, such as teleost fish, amphibians and birds. There are genes coding for lamin A (no splicing to the lamin C variant), lamin B1 (L1), lamin B2 (LII) and lamin B3 (LIII), the last of which is only expressed in oocytes and the early embryo. In amphibians, the lamin B3 gene codes for three alternatively spliced transcript products: lamin B3a (LIIIa), lamin B3b (LIIIb) and LIV [20]. Additional lamin B3 is also present in chickens, while some fish species have an additional gene for lamin A [22].

**Fig. 2:**
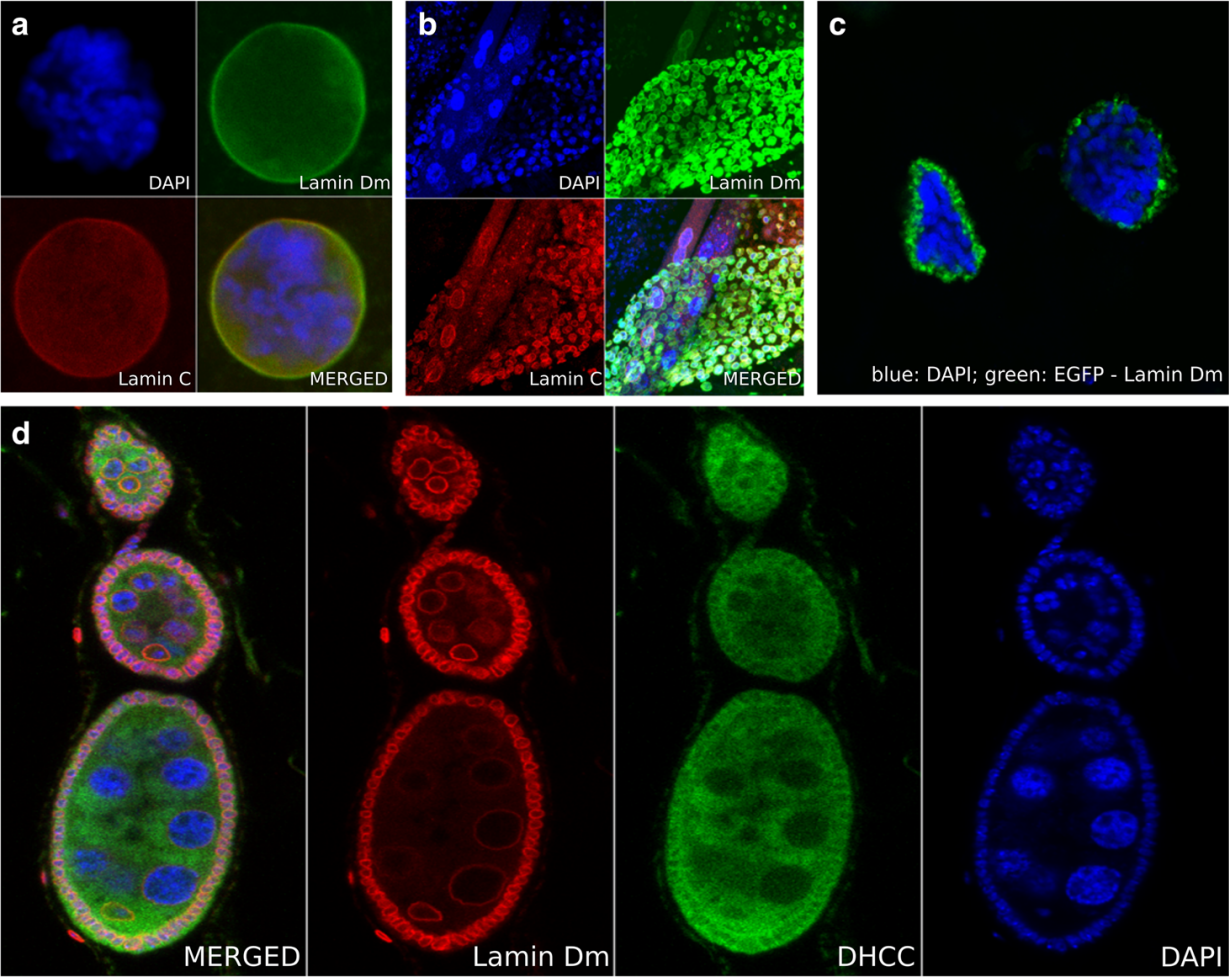
**a** The salivary gland nuclei of 3rd instar larvae with polytene chromosomes are the best known feature of the *Drosophila* model system. Dissected salivary glands and prepared nuclei are shown, stained for lamin Dm (green) with rabbit affinity purified anti-lamin Dm antibody and lamin C (red) with the ALC28.12 monoclonal antibody. DNA is counterstained with DAPI. **b** A dissected 3rd instar larvae thick specimen stained for lamin Dm (green) and lamin C (red) with antibodies as in section A. Only differentiated cells express lamin C. Both diploid and polyploid nuclei are visible. **c** Salivary gland nuclei of 3rd instar larvae, with polytene chromosomes, dissected from a fly strain overexpressing EGFP-lamin Dm (green) under the control of the Act5C-Gal4 driver. Note the increased thickness of the nuclear lamina and its irregular structure, composed of aggregates, cisterns and invaginations. **d** Nuclei of nurse cells of egg chambers and follicular cells stained for lamin Dm (red) and membranes (green). *Drosophila* egg chambers contain nurse cells with polyploid nuclei (up to 1024 N) and are frequently used as controls for chromatin organization in polyploid cells without polytene chromosome structure. Please note the diploid oocyte nuclei in which chromatin fills only part of the cell nucleus

The fly genome is unique in invertebrates: it has one gene for B-type lamin (lamin Dm) and one for A-type lamin (lamin C) [19, 23, 24]. Fig. 2a and b shows the typical staining pattern for lamin Dm and lamin C in larval tissues. Lamin Dm (green) is expressed in all tissues while lamin C (red) is only expressed in differentiated ones (Fig. 2b). Both proteins are located at the nuclear lamina. The major additional advantage of the fly model system is the giant, polytene (up to 1024 N) chromosomes (Fig. 2a) present in salivary gland cells of third instar larvae (Additional file 1: Video S1). These can be used to visualize events taking place at particular loci using a combination of FISH/RISH with confocal IF (e.g. [25, 26]). The fly system is also connected with polyploid nuclei of nurse cells in egg chambers reaching up to 1024 N in the nuclei of cells next to the oocyte (Fig. 2d). The chromatin organization of such nuclei may serve as an excellent control for chromatin organization in the polytene nuclei of salivary glands. Finally, the fly model system offers a large collection of strains for tissue-specific expression of proteins and siRNA using a large variety of strains with tissue selective Gal4 drivers [27]. Fig. 2c shows dissected salivary glands nuclei from a fly strain overexpressing GFP-lamin Dm under the control of the Act5C-Gal4 driver. This demonstrates the great potential and usefulness of the genetic system of *Drosophila*.

The minor, technical disadvantage of the genetic system of the fly model, compared to the *C. elegans* model [28] is the necessity to keep large stocks of living fly strains as a library, while worms can be kept frozen.

## *Drosophila* Lamin B (Dm)

The *Drosophila* lamin Dm, which is a B-type, is longer than human lamin B1 (622 versus 586 residues) and is of similar length to human lamin B2 (620 residues). Lamin Dm has a longer head domain: 59 versus 36 residues (45 and 23 residues to the N-terminal Cdk1 site) and more phosphorylation sites in this domain than the mammalian lamin B1. The fly lamin Dm head domain is similar in length to human lamin B2 (59 versus 41 residues) and the location of the N-terminal Cdk1 site is similar to that in lamin B1 (37 residues from N-terminus). Concomitant with the longer head domain, fly lamin Dm also has an additional, 10-residue spacer in the tail domain separating the C-terminal Cdk1 and PKC/PKA site from the S/TRAT/S sequence, which is evolutionarily conserved in B1-type lamins but absent in B2 and A-type lamins [18].

The fly lamin Dm, unlike another widely used invertebrate model system, *C. elegans* lamin (Ce-lamin), but similarly to mammalian B-type lamins, contains the Cdk1/cdc2 site flanking rod domains. It also contains a typical Ig-fold domain and its carboxy terminus is farnesylated and methylated at the CaaX motif [18].

Lamin Dm is expressed in almost all fly tissues. There are at least three lamin Dm isoforms, which can be distinguished by electrophoretic mobility and/or different staining using phosphorylation-dependent antibodies: Dm_1_ and Dm_2_ are the interphase isoforms, and Dm_mit_ is the major mitotic isoform [29]. The Dm_2_ isoform arises from Dm_1_ by phosphorylation on the N-terminal domain at around S [25, 29–34]. Both Dm_1_ and Dm_2_ interact with nucleic acids in vivo [35]. Dm_mit_ is soluble during mitosis, presumably due to phosphorylation at the Cdk1 sites [29, 32, 35–39]. In vitro, higher-order assemblies of lamin Dm can be depolymerized by Cdk1, PKC or PKA (protein kinase A) [40, 41].

Reduction in the expression levels of lamin Dm has been studied in vitro [36, 37, 42, 43] and in vivo [44–48]. Complete genetic knockout of the lamin Dm allele is only lethal at the larval stage. This is probably due to the large amounts of maternally deposited lamin Dm. A genetic approach to solve this issue through depletion of maternally expressed lamin showed that lamin Dm is necessary for the maintenance of proper egg polarity and embryonic development [45]. The germ line mutants have abnormal dorsal–ventral polarity of the oocyte and transcripts of the dorsal determinant Gurken fail to localize properly around the anterodorsal surface of the oocyte nucleus [45]. Partial lamin Dm gene deletions result in milder phenotypes [44, 48].

## *Drosophila* Lamin C

*Drosophila* lamin C has 16 more residues in its N-terminal head domain than vertebrate A-type lamins. Lamin C has also an extra spacer (17 residues) compared to human lamin A, between the C-terminal Cdk1 site and the unstructured region containing the evolutionarily conserved Akt/PKC site [18]. Fly lamin C has a similar length to human lamin A (640 versus 664 residues, out of which 14 are cleaved off post-translationally) but has a significantly shorter tail domain than human lamin A and therefore lacks two of several conservative motifs on its tail characteristic for vertebrate lamin A proteins. On the other hand, it is longer than vertebrate lamins C (640 versus 575 residues) and its tail domain is longer [49].

Lamin C also lacks a farnesylation motif on its tail. All other essential conservative motifs of A-type lamins are present, including two Cdk1 sites flanking the rod domain. Thus, it can be considered as an A-type lamin with more similarity to vertebrate lamin C than A [18]. Note that fly lamin C has its “SRATS” motif in its head domain instead of its tail domain. Since this motif is considered to be a part of the chromatin/DNA-binding motif [50] and is a a regulatory motif in the polymerization of lamins [35], its location may have an impact on lamin C properties [18].

Lamin C expression is regulated developmentally [51]. Overexpression of lamin C seems to be stage-specifically lethal [52], while genetic knockdown results in muscular defects and semi-lethality when only truncated, N-terminally deleted lamin C was expressed [53–56].

The presence of two lamin genes coding for lamin Dm and lamin C, which respectively correspond to B-type lamin and A-type lamin of vertebrates, makes the fly system a useful tool for studies of laminopathies. Although the fly lamin C gene originated from duplicated ancestor gene for lamin Dm, lamin C evolved to gain structural and functional similarity to lamin A-type proteins of vertebrates [18, 20, 21, 28, 49, 57].

Another advantage of the fly system is the in vitro nuclear assembly system widely used for studies of nuclear protein functions, taking advantage of fly embryo extracts or fly oocyte extracts or insect cells infected with the baculovirus system [36, 37, 42, 43, 58]. It is also possible to investigate fly lamin properties in *Xenopus* in vitro nuclear assembly or in *Xenopus* oocytes [35, 59].

An additional advantage of the fly system is that the fly genome encodes most of the lamin-interacting proteins characteristic for vertebrates, including some that have been experimentally verified: LINC complex components [60], dLBR (lamin B receptor) [61], and the LEM-domain proteins otefin [62, 63], bocksbeutel (CG9424) isoforms [64], *Drosophila* MAN1 isoforms [65–67] and BAF (barrier-to-autointegration factor) [68, 69]. Additional file 2: Figure S1 shows multiple sequence alignment for BAF protein, demonstrating the very high similarity between BAF proteins from *C. elegans* and humans. Similar multiple sequence alignments for LEM-domain proteins MAN1 and LBR show the very low similarity between these proteins in *C. elegans* and in *Drosophila* or human (Additional files 3 and 4: Figures S2 and S3). MAN1 protein (single protein) in the fly system is translated from 3 different transcripts: A, B and C. There are also three transcripts for LBR protein in flies, which can give rise to two translation products: isoforms A and B, which are identical, and isoform C which has a longer N-terminus.

## Laminopathy model based on Lamin Dm

Several genetic models have been tested for the function of the lamin Dm proteins. After the initial discoveries of lamin Dm null lethality and the role of maternally deposited lamin Dm in vivo [45, 46], studies focused on the discrete mechanisms underlying this lethality. Lamin Dm knockout or siRNA-mediated knockdown result in reorganization and transcriptional activation of heterochromatic, testis-specific gene clusters in somatic tissues. These were also associated with the detachment of these loci from their nuclear envelope location [70]. This suggests that lamin Dm plays an important role in chromatin organization and heterochromatin maintenance. Genetic studies on the role of lamin Dm on position effect variegation using the lamin Dm Ari allele (the farnezylation-deficient mutant of lamin Dm) and wm4 allele revealed a strong silencing effect [71]. Further analyses demonstrated that lamin Dm null neuroblasts proliferate normally (no lamin C expressed in the neuroblasts), but further development of gonad and CNS (central nervous system) tissues demonstrated hypertrophy but hypotrophy of the ventriculus and muscle layer formation was observed. The latter was associated with a decreased level of ecdysteroid hormone receptor (EcRB1) protein [44, 48].

The fly system was also used to test aging related to lamins. It was demonstrated that age-related loss of expression of lamin Dm in the fat body, a major immunomodulatory organ, results in immunosenescence, which induces strong systemic inflammation. This in turn induces hyperplasia in the midgut. Lamin Dm loss also induces heterochromatin loss in fat body cells and de-repression of immune response genes [72].

Another aging-related phenotype can be observed in flies with overexpression of lamin Dm and/or kugelkern proteins [73]. Both proteins contain farnesylation motifs on the C-terminus. Thus, the phenotype may be a specific result of protein overexpression or an unspecific effect related to induction of nuclear envelope blebbing by additional amounts of farnesylated proteins, since overexpression of a C-terminal fragment of farnesylated protein also causes NE increase and blebbing. Another fly study demonstrated that through interaction with Nup107, lamin Dm is involved in proper organization of spindle microtubules during male meiosis [74].

## Laminopathy model based on fly Lamin C

The lamin C gene is located within intron 5 of the essential fly gene *tout velu* (ttv), which caused considerable problems with genetic analyses of lamin C function. Nevertheless, the first systematic study of the gene using stage-specific expression of siRNA demonstrated a role for lamin C in development related to its effect on chromatin organization through relocation of HP1 protein. Furthermore, the lamin C mutant suppressed position effect variegation. Overexpression of lamin C was found to be stage-specifically lethal through induction of caspase-dependent apoptosis [54]. Stage-specific lethality induced by ectopic expression of lamin C was confirmed independently [52] suggesting that the lamin C protein is necessary for fly development. Surprisingly, the fly nervous system was not affected when lamin C expression was specifically targeted to this tissue [54]. Another study, using the GAL4-UAS genetic system, tested the effect of ectopic expression of lamin C in the larval body wall muscles, showing no visible phenotype [55].

When an N-terminally truncated version of lamin C lacking the first 48 N-terminal residues, including the Cdk1 site (lamin C delta N), was expressed, the semi-lethality phenotype was observed. Survivors in adulthood demonstrated leg muscle atrophy and dysregulated hormone regulatory pathways [53]. This indicates that fly lamin C not only structurally but also functionally resembles vertebrate lamin A/C.

The first systemic, comparable studies of fly lamin C and human lamin A in the fly system were performed to test the fly model as a tool for laminopathies. They demonstrated that loss of lamin C results in nuclear envelope abnormalities resembling vertebrate lamin A loss. Ectopic expression of analogs of human laminopathic lamin A mutations in fly lamin C protein also resulted in muscle abnormalities typical for the muscular laminopathy phenotype as in humans [55] Further studies demonstrated that fly lamin C loss resulted in pupal metamorphic lethality. This effect was assigned specifically to the abnormal phenotype in tendon cells. There was a complete loss of organization of shortstop protein, belonging to the spectraplakin family, around the cell nucleus. The wild-type phenotype was restored when lamin C was expressed in tendon cells but not when lamin C was expressed in skeletal muscle cells [56].

Preliminary data on nuclear stretching in the stretched larval body wall muscles expressing the headless lamin C mutant indicated that intact N-terminal lamin C (head domain) is necessary for proper strain resistance [75].

## Human laminopathy mutations tested in the *Drosophila* model

Since the fly model offers a unique opportunity to design simple genetic systems for genetic analyses of the function of particular protein, it was also used to study human intermediate filament (IF) properties, including those of lamins and lamina-associated proteins [76–80] The initial study simply compared the location and the effect of the expression of human lamin A, C, B1 and B2 in *Drosophila* larval tissue to the location of endogenous fly lamin Dm and C [55]. Human lamins generally localized to the nuclear lamina, similarly to the endogenous lamins, but human lamin C was more nucleoplasmic than fly lamin C. Human lamin B2 frequently formed extra envelope structures, especially when overexpressed. Yeast two-hybrid system studies demonstrated evolutionary conservation of interactions between fly and human lamins and LEM-domain proteins.

Fly lamin C null cells showed nuclear envelope defects similarly to the human lamin A phenotype in mammals. Expression of fly lamin C with mutations representing human lamin A laminopathy mutations such as N210K (N195K in human lamin A/C), R401K (R386K), K493W (K453W), W557S (W520S) and L567P (L530P) under the control of different drivers were lethal except when drivers provided a low level of mutant protein comparable to endogenous lamin C [55, 79].

Another study demonstrated the effect of the expression of a new set of laminopathy mutations – G489 V (G449 V), N496I (N456I), V528P (L489P) and M553R (W514R) – in the body wall muscles of fly larvae [75, 78]. These mutants were also mostly lethal when expression was driven by the Mef2 promoter (embryo and larvae specific) and viable when MHC promoter (adult muscle specific) was used. Some of the mutants partially relocated a fraction of FG-repeat nucleoporins, gp210 protein and klaroid protein.

In another study, mutants and headless fly lamin C were shown to affect the expression of genes using total RNA isolation and microarray (Drosophila 2.0 GeneChip array) [81]. The expression of the G489 V mutant changes the expression of 87 genes compared to the wild type, while headless lamin C affected the expression of 28 genes. Of these two sets of genes, there was an overlap of 21 genes affected coding for proteins involved in large variety of functions. Two of them (glutathione transferase and oxidoreductase) were associated with oxidative/reductive stress [81]. Nuclear translocation of Cap-and-collar-C protein, a fly homologue of human Nrf2 protein, was observed, as was the disappearance of Keep1 proteins. This suggests that the Nrf2 pathway may contribute to the toxicity of laminopathy mutations V528P and M553R.

Our knowledge suggests that the *Drosophila melanogaster* model system for studies of nucleus biology and the functions of nuclear proteins, especially lamins and lamina-associated proteins, reflects very well processes from vertebrates and mammals. Therefore, the fly model system seems to be a very attractive animal model system for the study of lamins, laminopathies and a large variety of other genetic disorders and can contribute considerable valuable data impossible to generate in vertebrates.

## Additional file

Additional file 1: **Video S1:** The video shows a 3D model of salivary gland nuclei stained for lamin Dm (red) with monoclonal antibodies ADL67.12 and stained for DNA with DAPI to visualize polytene chromosomes. Please note that the contacts between lamin Dm protein and the chromosomes are not limited to nuclear rim/nuclear lamina/NE but lamin Dm “intercalates” in between polytene chromosomes. (AVI 84131 kb)

Additional file 2: **Figure S1.** Multiple sequence alignment of BAF proteins from different species: *Caenorhabditis elegans* (NP_499085.1 Barrier-to-autointegration factor 1), *Drosophila melanogaster* (NP_001260220.1 barrier to autointegration factor), *Xenopus laevis*: factor A (NP_001084558.1 barrier-to-autointegration factor A) and factor B (NP_001087314.1 barrier-to-autointegration factor B), *Mus musculus* (NP_001033320.1 barrier-to-autointegration factor), and *Homo sapiens* (NP_003851.1 barrier-to-autointegration factor). The darker blue color indicates higher similarity. The numbering above the multiple sequence alignment is for *D. melanogaster*. All sequences were aligned with CLUSTALX v. 2.0. Each alignment was edited in JALVIEW v. 2.8. and individually corrected for inaccurate fragments. (PNG 33 kb)

Additional file 3: **Figure S2.** Multiple sequence alignment of MAN1 proteins from different species: *Caenorhabditis elegans* (NP_496944.1 LEM protein 2), *Drosophila melanogaster* (NP_001286812.1 MAN1), *Xenopus laevis*: (NP_001082578.1 LEM domain containing 3 S homeolog), *Mus musculus* (NP_001074662.2 inner nuclear membrane protein Man1), and *Homo sapiens*: isoform 1 (NP_055134.2 inner nuclear membrane protein Man1 isoform 1) and isoform 2 (NP_001161086.1 inner nuclear membrane protein Man1 isoform 2). The darker blue color indicates higher similarity. The numbering above the multiple sequence alignment is for *D. melanogaster*. All sequences were aligned with CLUSTALX v. 2.0. Each alignment was edited in JALVIEW v. 2.8. and individually corrected for inaccurate fragments. (PNG 309 kb)

Additional file 4: **Figure S3.** Multiple sequence alignment of LBR proteins from different species: *Drosophila melanogaster*: isoform A (NP_611608.1 lamin B receptor, isoform A), isoform B (NP_726115.1 lamin B receptor, isoform B), isoform C (NP_726114.1 lamin B receptor, isoform C), *Xenopus laevis* (NP_001079301.1 lamin B receptor S homeolog), *Mus musculus* (NP_598576.2 lamin-B receptor), and *Homo sapiens* (NP_002287.2 lamin-B receptor). Isoforms A and B of LBR proteins in *D. melanogaster* are shown on one alignment because they have the same amino acid sequence. The darker blue color indicates higher similarity. The numbering above the multiple sequence alignment is for *D. melanogaster*. All sequences were aligned with CLUSTALX v. 2.0. Each alignment was edited in JALVIEW v. 2.8. and individually corrected for inaccurate fragments. (PNG 217 kb)

## Abbreviations

BAF: Barrier-to-autointegration factor
CNS: Central nervous system
INM: Inner nuclear membrane
LBR: Lamin-B receptor
LINC complex: Linker of nucleoskeleton and cytoskeleton complex
MTOC: Microtubule-organizing center
NE: Nuclear envelope
NEBD: Nuclear envelope breakdown
NL: Nuclear lamina
NLS: Nuclear localization signal
NPC: Nuclear pore complex
ONM: Outer nuclear membrane

## References

[1] Gruenbaum Y, Foisner R (2015). Lamins: nuclear intermediate filament proteins with fundamental functions in nuclear mechanics and genome regulation. Annu Rev Biochem.

[2] Turgay Y, Eibauer M, Goldman AE, Shimi T, Khayat M, Ben-Harush K (2017). The molecular architecture of lamins in somatic cells. Nature.

[3] Turgay Y, Medalia O (2017). The structure of Lamin filaments in somatic cells as revealed by cryo-electron tomography. Nucleus.

[4] Rzepecki R (2002). The nuclear lamins and the nuclear envelope. Cell Mol Biol Lett.

[5] Ruan J, Xu C, Bian C, Lam R, Wang JP, Kania J (2012). Crystal structures of the coil 2B fragment and the globular tail domain of human Lamin B1. FEBS Lett.

[6] Machowska M, Piekarowicz K, Rzepecki R. Regulation of Lamin properties and functions: does phosphorylation do it all? Open Biology. 2015;5:150094. 10.1098/rsob.150094. 10.1098/rsob.150094 PMC4680568 26581574

[7] Bronshtein I, Kepten E, Kanter I, Berezin S, Lindner M, Redwood AB (2015). Loss of Lamin a function increases chromatin dynamics in the nuclear interior. Nat Commun.

[8] Shimi T, Pfleghaar K, Kojima SI, Pack CG, Solovei I, Goldman AE (2008). The A- and B-type nuclear Lamin networks: microdomains involved in chromatin organization and transcription. Genes Dev.

[9] Spann TP, Goldman AE, Wang C, Huang S, Goldman RD (2002). Alteration of nuclear Lamin organization inhibits RNA polymerase II-dependent transcription. J Cell Biol.

[10] Kumaran RI, Muralikrishna B, Parnaik VK (2002). Lamin a/C speckles mediate spatial organization of splicing factor compartments and RNA polymerase II transcription. J Cell Biol.

[11] Stephens AD, Banigan EJ, Adam SA, Goldman RD, Marko JF (2017). Chromatin and Lamin a determine two different mechanical response regimes of the cell nucleus. Mol Biol Cell.

[12] Patterson K, Molofsky AB, Robinson C, Acosta S, Cater C, Fischer JA (2004). The functions of klarsicht and nuclear Lamin in developmentally regulated nuclear migrations of photoreceptor cells in the Drosophila eye. Mol Biol Cell.

[13] Mattioli E, Columbaro M, Capanni C, Maraldi NM, Cenni V, Scotlandi K (2011). Prelamin A-mediated recruitment of SUN1 to the nuclear envelope directs nuclear positioning in human muscle. Cell Death Differ.

[14] Meinke P, Mattioliz E, Haque F, Antoku S, Columbaro M, Straatman KR (2014). Muscular dystrophy-associated SUN1 and SUN2 variants disrupt nuclear-cytoskeletal connections and Myonuclear organization. PLoS Genet.

[15] Buxboim A, Swift J, Irianto J, Spinler KR, Dingal PC, Athirasala A (2014). Matrix elasticity regulates Lamin-a,C phosphorylation and turnover with feedback to actomyosin. Curr Biol.

[16] Harada T, Swift J, Irianto J, Shin JW, Spinler KR, Athirasala A (2014). Nuclear Lamin stiffness is a barrier to 3D migration, but softness can limit survival. J Cell Biol.

[17] Swift J, Ivanovska IL, Buxboim A, Harada T, Dingal PC, Pinter J (2013). Nuclear Lamin-a scales with tissue stiffness and enhances matrix-directed differentiation. Science.

[18] Rzepecki R. The nuclear lamins and nuclear envelope. Cell Mol Biol Letters. 2002;7:1019–35. 12511969

[19] Melcer S, Gruenbaum Y, Krohne G (2007). Invertebrate lamins. Exp Cell Res.

[20] Peter A, Stick R (2012). Evolution of the Lamin protein family: what introns can tell. Nucleus.

[21] Stick R, Peter A (2017). Evolutionary changes in Lamin expression in the vertebrate lineage. Nucleus.

[22] Dechat T, Pfleghaar K, Sengupta K, Shimi T, Shumaker DK, Solimando L (2008). Nuclear lamins: major factors in the structural organization and function of the nucleus and chromatin. Genes Dev.

[23] Bossie CA, Sanders MM (1993). A cDNA from Drosophila melanogaster encodes a Lamin C-like intermediate filament protein. J Cell Sci.

[24] Gruenbaum Y, Landesman Y, Drees B, Bare JW, Saumweber H, Paddy MR (1988). Drosophila nuclear Lamin precursor Dm0 is translated from either of two developmentally regulated mRNA species apparently encoded by a single gene. J Cell Biol.

[25] Capelson M, Doucet C, Hetzer MW (2010). Nuclear pore complexes: guardians of the nuclear genome. Cold Spring Harb Symp Quant Biol.

[26] Capelson M, Liang Y, Schulte R, Mair W, Wagner U, Hetzer MW (2010). Chromatin-bound nuclear pore components regulate gene expression in higher eukaryotes. Cell.

[27] Caygill EE, Brand AH. The GAL4 System: A Versatile System for the Manipulation and Analysis of Gene Expression (vol 1478, pg 33, 2016). Drosophila: Methods and Protocols, 2nd Edition 2016; 1478:E1–E3. 10.1007/978-1-4939-6371-3_2 27730574

[28] Rzepecki R, Gruenbaum Y. Invertebrate model of Lamin diseases. Nucleus 2018;9(1):227-234. 10.1080/19491034.2018.1454166 PMC5973256 29557730

[29] Rzepecki R, Fisher PA (2002). In vivo phosphorylation of Drosophila melanogaster nuclear lamins during both interphase and mitosis. Cell Mol Biol Lett.

[30] Lin L, Fisher PA (1990). Immunoaffinity purification and functional characterization of interphase and meiotic Drosophila nuclear Lamin isoforms. J Biol Chem.

[31] McConnell M, Whalen AM, Smith DE, Fisher PA (1987). Heat shock-induced changes in the structural stability of proteinaceous karyoskeletal elements in vitro and morphological effects in situ. JCell Biol.

[32] Smith DE, Fisher PA (1989). Interconversion of Drosophila nuclear Lamin isoforms during oogenesis, early embryogenesis, and upon entry of cultured cells into mitosis. J Cell Biol.

[33] Smith DE, Gruenbaum Y, Berrios M, Fisher PA (1987). Biosynthesis and interconversion of Drosophila nuclear Lamin isoforms during normal growth and in response to heat shock. J Cell Biol.

[34] Zaremba-Czogalla M, Gagat P, Koziol K, Dubinska-Magiera M, Sikora J, Dadlez M (2011). Identification of new in vivo phosphosites on Lamin Dm-the evidence of heterogeneity of phosphorylation sites in different Drosophila tissues. Nucleus-Austin.

[35] Zaremba-Czogalla M, Piekarowicz K, Wachowicz K, Koziol K, Dubinska-Magiera M, Rzepecki R (2012). The different function of single phosphorylation sites of Drosophila melanogaster Lamin Dm and Lamin C. PLoS One.

[36] Fisher PA, Berrios M. Cell-free nuclear assembly and disassembly in Drosophila. Methods Cell Biol. 1998:397–416. 10.1016/s0091-679x(08)60888-2 9348518

[37] Maus N, Stuurman N, Fisher PA (1995). Disassembly of the Drosophila nuclear lamina in a homologous cell-free system. J Cell Sci.

[38] Stuurman N, Maus N, Fisher PA (1995). Interphase phosphorylation of the Drosophila nuclear Lamin: site-mapping using a monoclonal antibody. J Cell Sci.

[39] Stuurman N, Sasse B, Fisher PA (1996). Intermediate filament protein polymerization: molecular analysis of Drosophila nuclear Lamin head-to-tail binding. J Struct Biol.

[40] Stuurman N (1997). Identification of a conserved phosphorylation site modulating nuclear Lamin polymerization. FEBS Lett.

[41] Zaremba-Czogalla M, Gagat P, Koziol K, Dubinska-Magiera M, Sikora J, Dadlez M (2011). Identification of new in vivo phosphosites on Lamin Dm - the evidence of heterogeneity of phosphorylation sites in different Drosophila tissues. Nucleus.

[42] Ulitzur N, Harel A, Feinstein N, Gruenbaum Y (1992). Lamin activity is essential for nuclear envelope assembly in a Drosophila embryo cell-free extract. J Cell Biol.

[43] Ulitzur N, Harel A, Goldberg M, Feinstein N, Gruenbaum Y (1997). Nuclear membrane vesicle targeting to chromatin in a Drosophila embryo cell-free system. Mol Biol Cell.

[44] Furukawa K, Osouda S, Sugiyama S, Horigome T, Fisher P (2005). Null mutants of Drosophila B-type Lamin Dm0 show aberrant tissue differentiation rather than obvious nuclear shape distortion or specific defects during cell proliferation. Mech Develop.

[45] Guillemin K, Williams T, Krasnow MA (2001). A nuclear Lamin is required for cytoplasmic organization and egg polarity in Drosophila. Nat Cell Biol.

[46] Lenz-Bohme B, Wismar J, Fuchs S, Reifegerste R, Buchner E, Betz H (1997). Insertional mutation of the Drosophila nuclear Lamin Dm0 gene results in defective nuclear envelopes, clustering of nuclear pore complexes, and accumulation of annulate lamellae. J Cell Biol.

[47] Osouda S, Horigome T, Sugiyama S, MoConnell M, Fisher PA, Furukawa K (2004). Loss of Drosophila Lamin Dm0 induces late pupal/early adult lethality and defective differentiation accompanied by a decrease in ecdysteroid hormone receptor. Mol Biol Cell.

[48] Osouda S, Nakamura Y, de Saint Phalle B, McConnell M, Horigome T, Sugiyama S (2005). Null mutants of Drosophila B-type Lamin Dm(0) show aberrant tissue differentiation rather than obvious nuclear shape distortion or specific defects during cell proliferation. Dev Biol.

[49] Riemer D, Weber K (1994). The organization of the gene for Drosophila Lamin C: limited homology with vertebrate Lamin genes and lack of homology versus the Drosophila Lamin Dm0 gene. Eur JCell Biol.

[50] Goldberg M, Harel A, Brandeis M, Rechsteiner T, Richmond TJ, Weiss AM (1999). The tail domain of Lamin Dm0 binds histones H2A and H2B. Proc Natl Acad Sci U S A.

[51] Riemer D, Stuurman N, Berrios M, Hunter C, Fisher PA, Weber K (1995). Expression of Drosophila Lamin C is developmentally regulated: analogies with vertebrate A-type lamins. J Cell Sci.

[52] Stuurman N, Delbecque JP, Callaerts P, Aebi U (1999). Ectopic overexpression of Drosophila Lamin C is stage-specific lethal. Exp Cell Res.

[53] Dialynas G, Speese S, Budnik V, Geyer PK, Wallrath LL (2010). The role of Drosophila Lamin C in muscle function and gene expression. Development.

[54] Gurudatta BV, Shashidhara LS, Parnaik VK (2010). Lamin C and chromatin organization in Drosophila. J Genet.

[55] Schulze SR, Curio-Penny B, Li Y, Imani RA, Rydberg L, Geyer PK (2005). Molecular genetic analysis of the nested Drosophila melanogaster Lamin C gene. Genetics.

[56] Uchino R, Nonaka YK, Horigome T, Sugiyama S, Furukawa K (2013). Loss of Drosophila A-type Lamin C initially causes tendon abnormality including disintegration of cytoskeleton and nuclear lamina in muscular defects. Dev Biol.

[57] Schilf P, Peter A, Hurek T, Stick R (2014). Lamins of the sea lamprey (Petromyzon marinus) and the evolution of the vertebrate Lamin protein family. Eur J Cell Biol.

[58] Klapper M, Exner K, Kempf A, Gehrig C, Stuurman N, Fisher PA (1997). Assembly of A- and B-type lamins studied in vivo with the baculovirus system. J Cell Sci.

[59] Grossman E, Dahan I, Stick R, Goldberg MW, Gruenbaum Y, Medalia O (2012). Filaments assembly of ectopically expressed Caenorhabditis elegans Lamin within Xenopus oocytes. J Struct Biol.

[60] Shuoshuo W, Adriana R, Talila V. Nesprin provides elastic properties to muscle nuclei by cooperating with spectraplakin and EB1. J Cell Biol. 2015;209:(4)529–38. 10.1083/jcb.201408098 PMC4442817 26008743

[61] Wagner N, Weber D, Seitz S, Krohne G (2004). The Lamin B receptor of Drosophila melanogaster. J Cell Sci.

[62] Ashery-Padan R, Ulitzur N, Arbel A, Goldberg M, Weiss AM, Maus N (1997). Localization and posttranslational modifications of otefin, a protein required for vesicle attachment to chromatin, during Drosophila melanogaster development. Mol Cell Biol.

[63] Goldberg M, Lu HH, Stuurman N, Ashery-Padan R, Weiss AM, Yu J (1998). Interactions among Drosophila nuclear envelope proteins Lamin, otefin, and YA. Mol Cell Biol.

[64] Wagner N, Schmitt J, Krohne G (2004). Two novel LEM-domain proteins are splice products of the annotated Drosophila melanogaster gene CG9424 (Bocksbeutel). Eur J Cell Biol.

[65] Wagner N, Kagermeier B, Loserth S, Krohne G (2006). The Drosophila melanogaster LEM-domain protein MAN1. Eur J Cell Biol.

[66] Wagner N, Krohne G (2007). LEM-domain proteins: new insights into Lamin-interacting proteins. Int Rev Cytol.

[67] Barton LJ, Wilmington SR, Martin MJ, Skopec HM, Lovander KLE, Pinto BS (2014). Unique and shared functions of nuclear Lamina LEM domain proteins in Drosophila. Genetics.

[68] Furukawa K (1999). LAP2 binding protein 1 (L2BP1/BAF) is a candidate mediator of LAP2-chromatin interaction. J Cell Sci.

[69] Furukawa K, Sugiyama S, Osouda S, Goto H, Inagaki M, Horigome T (2003). Barrier-to-autointegration factor plays crucial roles in cell cycle progression and nuclear organization in Drosophila. J Cell Sci.

[70] Shevelyov YY, Lavrov SA, Mikhaylova LM, Nurminsky ID, Kulathinal RJ, Egorova KS (2009). The B-type Lamin is required for somatic repression of testis-specific gene clusters. Proc Natl Acad Sci U S A.

[71] Bao XM, Girton J, Johansen J, Johansen KM (2007). The Lamin Dm(0) allele Ari3 acts as an enhancer of position effect variegation of the w (m4) allele in Drosophila. Genetica.

[72] Chen ZJ, Wang WP, Chen YC, Wang JY, Lin WH, Tai LA (2014). Dysregulated interactions between Lamin a and SUN1 induce abnormalities in the nuclear envelope and endoplasmic reticulum in progeric laminopathies. J Cell Sci.

[73] Brandt A, Krohne G, Grossans J (2008). The farnesylated nuclear proteins KUGELKERN and LAMIN B promote aging-like phenotypes in Drosophila flies. Aging Cell.

[74] Hayashi D, Tanabe K, Katsube H, Inoue YH (2016). B-type nuclear Lamin and the nuclear pore complex Nup107-160 influences maintenance of the spindle envelope required for cytokinesis in Drosophila male meiosis. Biol Open.

[75] Zwerger M, Jaalouk DE, Lombardi ML, Isermann P, Mauermann M, Dialynas G (2013). Myopathic Lamin mutations impair nuclear stability in cells and tissue and disrupt nucleo-cytoskeletal coupling. Hum Mol Genet.

[76] Bohnekamp J, Cryderman DE, Paululat A, Baccam GC, Wallrath LL, Magin TM (2015). A Drosophila model of epidermolysis bullosa simplex. J Investig Dermatol.

[77] Bohnekamp J, Cryderman DE, Thiemann DA, Magin TM, Wallrath LL (2016). Using Drosophila for studies of intermediate filaments. Method Enzymol.

[78] Dialynas G, Flannery KM, Zirbel LN, Nagy PL, Mathews KD, Moore SA (2012). LMNA variants cause cytoplasmic distribution of nuclear pore proteins in Drosophila and human muscle. Hum Mol Genet.

[79] Schulze SR, Curio-Penny B, Speese S, Dialynas G, Cryderman DE, McDonough CW (2009). A comparative study of Drosophila and human A-type lamins. PLoS One.

[80] Wallrath LL, Bohnekamp J, Magin TM (2016). Cross talk between the cytoplasm and nucleus during development and disease. Curr Opin Genet Dev.

[81] Dialynas G, Shrestha OK, Ponce JM, Zwerger M, Thiemann DA, Young GH (2015). Myopathic Lamin mutations cause reductive stress and activate the Nrf2/Keap-1 pathway. PLoS Genet.

