# Supplementary figures and images for: Laminopathies: what can humans learn from fruit flies
Source: Cell Mol Biol Lett. 2018 Jul 6;23:32. doi: 10.1186/s11658-018-0093-1 (PMC6034310; doi:10.1186/s11658-018-0093-1)

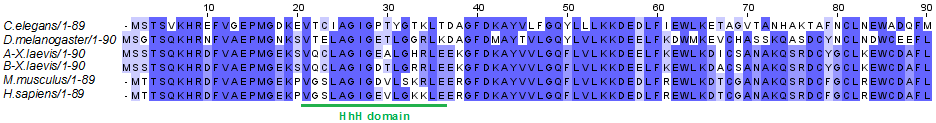

Supplement: Supplementary file 2 — Figure S1. Multiple sequence alignment of BAF proteins from different species: Caenorhabditis elegans (NP_499085.1 Barrier-to-autointegration factor 1), Drosophila melanogaster (NP_001260220.1 barrier to autointegration factor), Xenopus laevis: factor A (NP_001084558.1 barrier-to-autointegration factor A) and factor B (NP_001087314.1 barrier-to-autointegration factor B), Mus musculus (NP_001033320.1 barrier-to-autointegration factor), and Homo sapiens (NP_003851.1 barrier-to-autointegration factor). The darker blue color indicates higher similarity. The numbering above the multiple sequence alignment is for D. melanogaster. All sequences were aligned with CLUSTALX v. 2.0. Each alignment was edited in JALVIEW v. 2.8. and individually corrected for inaccurate fragments. (PNG 33 kb) [file 11658_2018_93_MOESM2_ESM.png]

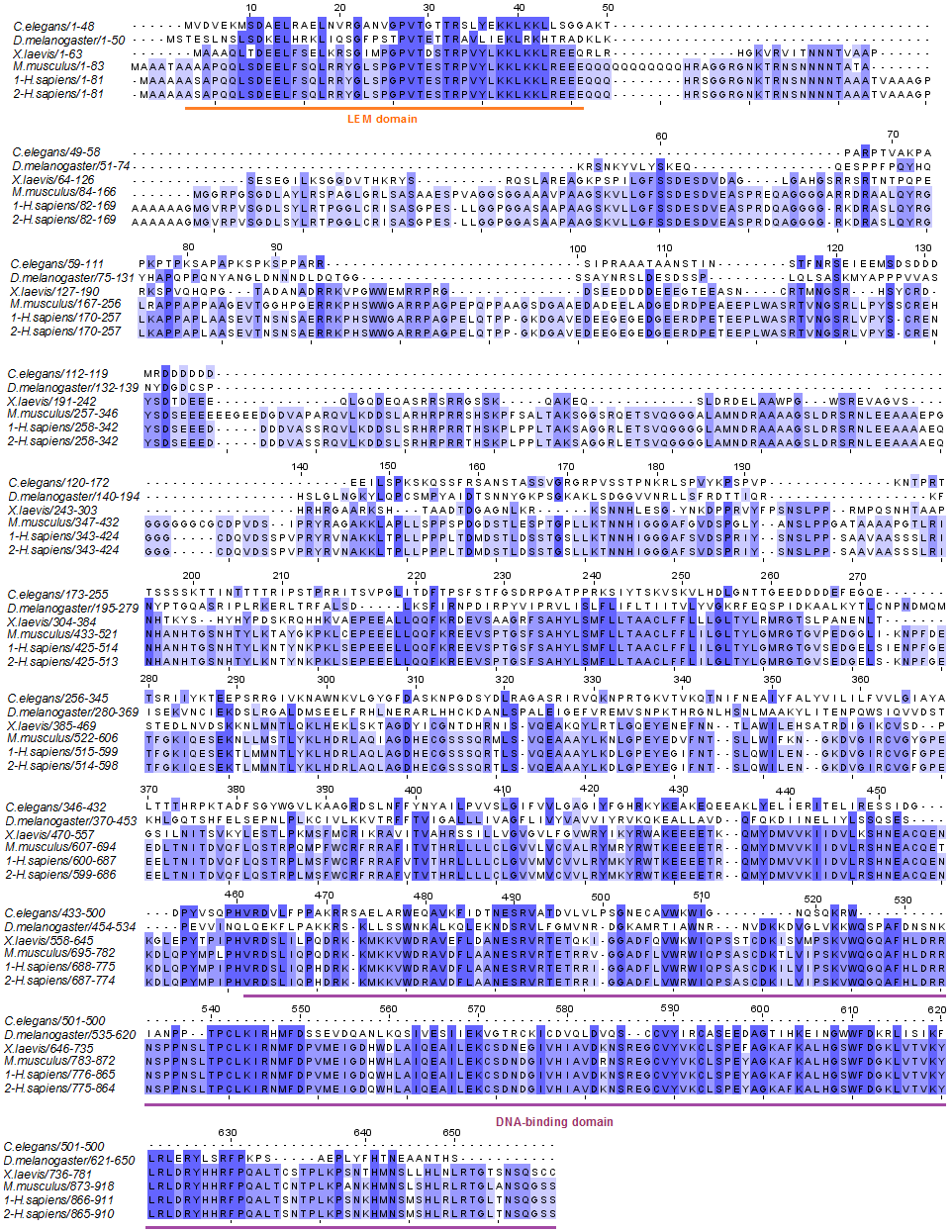

Supplement: Supplementary file 3 — Figure S2. Multiple sequence alignment of MAN1 proteins from different species: Caenorhabditis elegans (NP_496944.1 LEM protein 2), Drosophila melanogaster (NP_001286812.1 MAN1), Xenopus laevis: (NP_001082578.1 LEM domain containing 3 S homeolog), Mus musculus (NP_001074662.2 inner nuclear membrane protein Man1), and Homo sapiens: isoform 1 (NP_055134.2 inner nuclear membrane protein Man1 isoform 1) and isoform 2 (NP_001161086.1 inner nuclear membrane protein Man1 isoform 2). The darker blue color indicates higher similarity. The numbering above the multiple sequence alignment is for D. melanogaster. All sequences were aligned with CLUSTALX v. 2.0. Each alignment was edited in JALVIEW v. 2.8. and individually corrected for inaccurate fragments. (PNG 309 kb) [file 11658_2018_93_MOESM3_ESM.png]

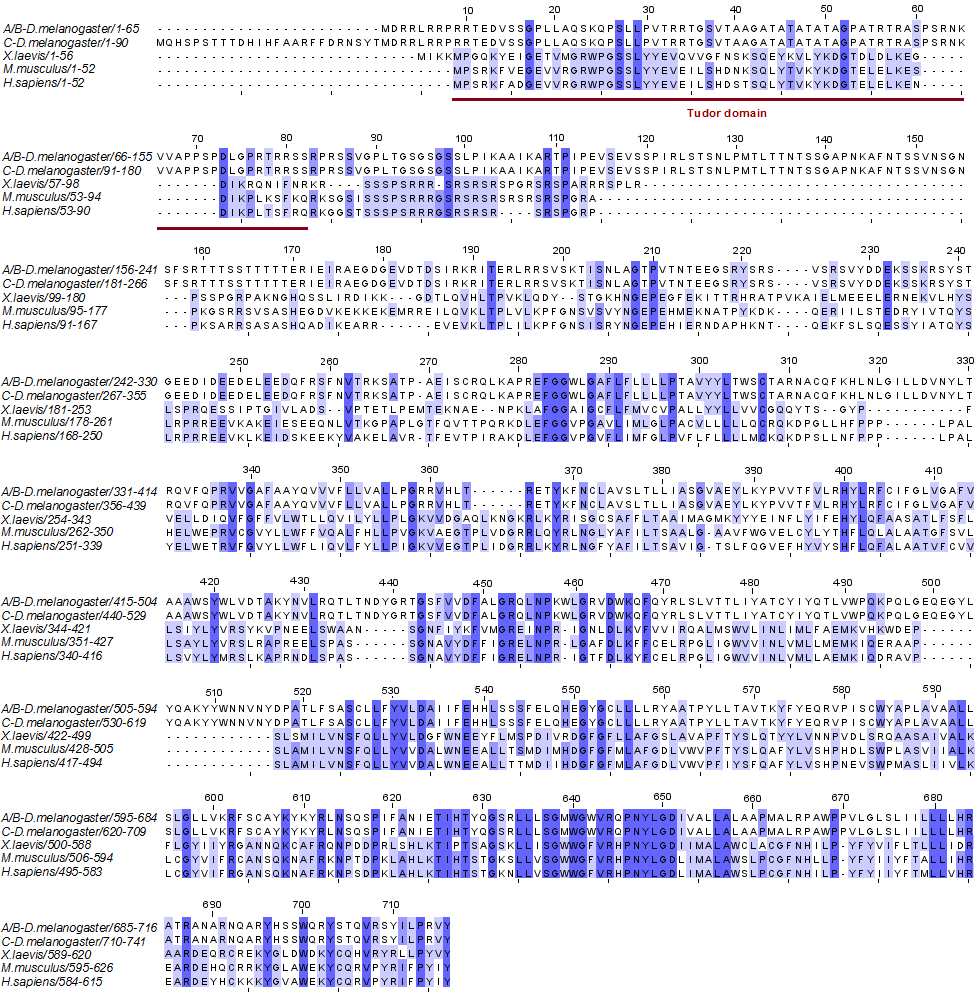

Supplement: Supplementary file 4 — Figure S3. Multiple sequence alignment of LBR proteins from different species: Drosophila melanogaster: isoform A (NP_611608.1 lamin B receptor, isoform A), isoform B (NP_726115.1 lamin B receptor, isoform B), isoform C (NP_726114.1 lamin B receptor, isoform C), Xenopus laevis (NP_001079301.1 lamin B receptor S homeolog), Mus musculus (NP_598576.2 lamin-B receptor), and Homo sapiens (NP_002287.2 lamin-B receptor). Isoforms A and B of LBR proteins in D. melanogaster are shown on one alignment because they have the same amino acid sequence. The darker blue color indicates higher similarity. The numbering above the multiple sequence alignment is for D. melanogaster. All sequences were aligned with CLUSTALX v. 2.0. Each alignment was edited in JALVIEW v. 2.8. and individually corrected for inaccurate fragments. (PNG 217 kb) [file 11658_2018_93_MOESM4_ESM.png]
